# Mechanisms in sociology—a critical intervention

**DOI:** 10.3389/fsoc.2024.1384979

**Published:** 2024-04-09

**Authors:** Dominik Döllinger

**Affiliations:** School of Humanities, Education and Social Sciences, Örebro University, Örebro, Sweden

**Keywords:** mechanisms, history of science, rationalization, critical theory, sociological theory

## Abstract

The notion of the mechanism is one of the most popular and widely used concepts in science and sociology is no exception. This paper problematizes the widespread and often uncritical use of the term “mechanism” in contemporary sociology. Drawing on the mechanistic worldview associated with leading figures of the scientific revolution, the paper emphasizes the impact of mechanistic thinking on the societal rationalization process identified by Max Weber and the Frankfurt School. The analysis suggests that mechanisms, when applied to sociological theories, may uncritically reproduce a cultural fetish of the rational society with potentially dehumanizing consequences. The author advocates for a critical reflection on the cultural and historical context of mechanisms, urging sociologists to view them not merely as analytical tools but as active contributors to the creation and shaping of social worlds erected on a belief in instrumental reason.

## 1 Introduction

Language matters in sociology. It matters how we talk about social issues, how we describe and frame social problems and, more generally, how we explain and theorize the social world. This implies that we constantly must reflect on the ideas and images that we invoke with our terminology. This is especially true when it comes to such popular concepts like the mechanism, a term that sociologists and other social scientists of the present day seem to use with great ease and often rather uncritically and without much consistency (Gerring, 2008; Ylikoski, 2018). Mechanisms were originally associated with the positivistic and pragmatist branches of sociology during the classical era, rose to theoretical prominence in the 1990s through the so-called social mechanisms program (Elster, 1989; Hedström and Swedberg, 1996, 1998; Hedström and Ylikoski, 2010; Edling and Rydgren, 2016), and nowadays find their way into research papers of both qualitative and quantitative sociologists of virtually any theoretical and methodological background. Glennan and Illari (2018) even argue that the social sciences were one of two major driving forces alongside several life sciences that are responsible for the resurgence of mechanistic philosophy in recent decades, and in articles that were recently published in this journal, sociologists reveal “different mechanisms of social network closure across generations” (Windzio and Kaminski, 2023), claim that “speech-acts can be seen as a mechanism to connect the brainpower of many individuals into a single collective power” (Van Langenhove, 2023), and find “preliminary evidence for an applied theory of culturally situated moral cognition as a coping mechanism with ethno-racial stress” (Firat, 2021).

Given that the notion of the mechanisms is maintaining or even increasing its popularity in sociology and the social sciences more broadly, we should also increase the amount of a critical reflection when it comes to their philosophical and practical implications. Here, it is interesting to note that there are numerous articles and books dedicated to the epistemological implications of mechanisms. Yet, it is quite difficult to find substantial discussions about the ontological, practical, and ethical implications of mechanistic thinking. This paper seeks to change this and inspire a debate about the use of mechanisms in sociology beyond their epistemological issues by framing them as a cultural fetish of the rationalized society. In doing so, I am not saying that mechanisms are by default a bad concept for sociologists. I am saying that there is a history and worldview attached to them that we need to be aware of before we use them in our research. If we decide to squeeze the social world into a mechanistic framework, this should be a conscious choice, not merely a scientistic habit of thought.

Throughout this paper, mechanisms are defined as the breakdown of processual reality into discrete analytical parts to explore their interactions, which produce effects of interest. The mechanistic worldview posits that the world can be meaningfully ordered and understood by creating analytical variables and establishing directions of influence between them. In sociology, this is often likened to opening a black box to examine how its composite parts interact (Hedström and Ylikoski, 2010; Ylikoski, 2018).

This definition of mechanisms is narrower than Gerring’s generic definition, which defines mechanisms as “the pathway or process by which an effect is produced” (Gerring, 2008, p. 161) and aligns more with the minimal definition presented by Glennan (2017) and Glennan and Illari (2018, p. 2), which treats mechanisms as “entities (or parts) whose activities and interactions are organized so as to be responsible for the phenomenon.” Gerring’s broad definition raises questions about what explanations would not be considered mechanistic and whether using the wording of the generic definition and eliminating the term “mechanism” altogether would reduce ambiguity and confusion even more.

Moreover, does the permissive use of mechanisms that Gerring outlines challenge critical examination of the concept. But neither a lack of consensus on the ontological and epistemological nature of mechanisms nor Gerring’s broad definition should render the concept immune to criticism. Just as a non-falsifiable hypothesis is not a good scientific hypothesis, a concept that cannot be critiqued due to its broadness and/or ambiguity, is not a particularly robust scientific concept, and being able to meet any critique of one’s conceptual apparatus by simply stating that this is not what one means when one uses the term, does not seem to be a particularly rigorous way forward. Bearing in mind the ongoing debates surrounding the ambivalence and broadness of the term, I will now turn to a sociological critique of mechanisms and mechanistic thinking based on the outlined understanding.

## 2 The mechanistic worldview

While mechanisms are older than modern science, most contemporary mechanistic philosophers and social scientists who entertain an understanding of mechanisms similar to the one a outlined above, associate themselves with the foundational thinkers of the scientific revolution like Hobbes, Descartes, Galileo, Boyle, Newton or Laplace, all of which proposed different version of a mechanical philosophy (see, e.g., Boas, 1952; Kuhn, 1996; Cook, 2001; Kochiras, 2013; Brown, 2023). Newton’s *Principica Mathematica*, for example, was an attempt to describe the physical world in mechanistic terms (though his concept of the force challenged parts of Descartes mechanical philosophy), and his rational mechanics reaffirmed the belief in a deterministic and mathematizable universe that could be understood as a complex machine operating according to fixed principles, a belief that had also been entertained by the aforementioned leading figures of the scientific revolution. During this latter revolution, even the human body was transformed into a machine. Descartes had already treated bodies in such a way, so much so that he famously equated animals—which he believed to have no soul—with physical machines (Descartes, 2006) and compared a dead human body to a broken watch (Descartes, 2015). The human body was essentially a machine, with the important exception that it was connected to (but not housing) a res cogitans (a thinking thing or soul) in the pineal gland. The idea of the body being a machine culminated in the idea of the *human machine* expressed by la Mettrie in 1747, which, despite initial criticism had anticipated later developments in physiology and psychology (Vartanian, 2015). Even as the emergence of quantum physics toward the end of the nineteenth century began to challenge the mechanistic outlook of Newtonian physics, it remained dominant in many of the scientific fields whose origins it had inspired. Today, mechanicism occupies a prominent place in biology, medicine, physiology and even psychology, especially within its neuro-scientific and cognitive-behavioral branches. Mechanicism was and continues to be one of science’s dominant worldviews. However, the implications of this mechanistic outlook, especially when applied to human beings, social interaction, and other social and social-psychological phenomena, has consequences that sociologists need to grapple with.

## 3 Mechanization and rationalization

From a sociological perspective, mechanicism contributes to the collective projection and universalization of the social ideals of an increasingly rationalized society on to the social, mental, and physical environment. The appreciation of mechanisms in science and society can be associated with the broader rationalization process that shaped modern industrialized societies, a trend that was already identified by Weber (1921, 1981) and further theorized by, among others, Ritzer (2015) and the theorists of the Frankfurt School. Even though the different theories regarding the rationalization of society focus on different aspects of this development, they generally agree that rationalization is the gradual transformation of (Western) societies during which traditional ways of living are replaced by formally rational institutions that emphasize economic efficiency, calculable and predictable outcomes, and environmental control. From the critical perspective of the Frankfurt School, rationalization describes the quasi-totalitarian takeover of instrumental reason as the predominant form of rationality since the Enlightenment (Horkheimer 1974; Adorno and Horkheimer, 1997; Horkheimer, 2002). Like Weber the Frankfurt School feared that society would turn into “a completely functional and antiseptic place” (Greisman and Ritzer, 1981) that was micromanaged by professionally trained bureaucrats and social engineers. Such a society that is erected on a belief in instrumental rationality with its calculable rules, technocratic administration and efficient procedures does, of course, need a science that provides the corresponding instrumental knowledge, that is, mechanistic knowledge. Weber, for example, describes that the peculiar Western form of capitalism and its rationality is:

“essentially dependent on the calculability of the most important technical factors. But this means fundamentally that it is dependent on the peculiarities of modern science, especially the natural sciences based on mathematics and exact and rational experiment” (Weber, 2001, p. xxxvii).

There is, in other words, an elective affinity between the previously mentioned mechanistic principles of the scientific revolution and the institutionalized ambition to explain, manipulate and control the environment with the help of applied scientific knowledge and technology in the rationalized society. Reasoning through mechanisms speaks to the affordances of a society shaped by instrumental reason with the ambition to provide unambiguous and predictable manipulation-tools that help us to productively and efficiently interfere in natural and social processes. As Horkheimer (2002, p. 194) observed:

“The manipulation of physical nature and of specific economic and social mechanisms demand alike the amassing of a body of knowledge such as is supplied in an ordered set of hypotheses. The technological advances of the bourgeois period are inseparably linked to this function of the pursuit of science.”

The search for mechanisms as the carving out of entities and the understanding of how their interactions produce certain effects is, then, one of the most promising ways of conducting science that is useful and integratable in a rational society, a process that is sometimes also referred to as scientization (Drori et al., 2003; Drori and Meyer, 2006).

The incorporation of instrumental reason through science can be observed on the structural and individual level. Within cognitive-behavioral psychotherapies, the dominant form of psychotherapy alongside medical treatments, therapists focus on observable behaviors, measurable outcomes (symptoms), and attempt to find specific techniques that manipulate the behavioral and cognitive patterns that are linked to these outcomes in order to bring about change in the most efficient way (Dobson and Dozois, 2010; Beck, 2011; Hofmann and Asmundson, 2017). This general mechanistic outlook of cognitive-behavioral therapy is exemplified in an article by David et al. (2018) with the title *Why Cognitive Behavioral Therapy Is the Current Gold Standard of Psychotherapy* in which the authors treat the terms model, theory, and mechanisms as synonyms as they discuss “underlying theories/mechanisms of change” (David et al., 2018: p. 2). This search for psychological mechanisms cannot be disentangled from the ambition to cure diseases and to manipulate the mental machine just like the emergence of the mechanical philosophy was meant be utilized to control, predict, and manipulate nature. In other words, the idea of science being the discoverer of mechanisms goes hand in hand with the idea of controlling and utilizing those mechanisms to improve conditions and cure diseases. However, as sociologists know full well, defining what counts as “improvement,” “cure” or “disease” is very much itself a constant negotiation and historical construction as has clearly been demonstrated by studies about the medicalization of society (Conrad, 2007), among others.

This intention of mechanisms to provide knowledge for control, manipulation and improvement can be extended to the type of mechanistic sociology that is discussed in this paper. When we describe or theorize a social phenomenon as a mechanism, we may—at least latently—(re)produce the rationalistic ambition to provide tools to manipulate and potentially improve it. However, not only is there no real objective way to measure “improvement” due to its historical embeddedness, mechanisms are generally lending themselves much easier to any ideology of improvement rather than fundamental structural change. Their inherent accumulative and non-dialectical character makes them favor the status quo over structural social change in the sense that they work toward the improvement of the existing under the parameters of the existing rather than toward a fundamental change of the existing conditions under which they operate. They expose weaknesses rather than contradictions. Hence, mechanisms are policy-friendly control-and manipulation-devices, sought after by politicians and social engineers to fix social problems, quite like how a cognitive-behavioral therapist wants to cure symptoms and behavioral patterns in an individual and a physiologist who wants to improve the condition of the human body under existing social conditions. Engineers of the body, the mind and society need mechanisms to work their magic. However, all these mechanistic outlooks cannot unfold the critical potential of dialectical thinking that sociology should hold dear.

Adorno (2000, p. 12) once stated that:

“[s]ociology […] has always had something technocratic about it, something of social engineering, a belief that if scientific experts, who make use of certain methodological techniques, are entrusted with the direct or indirect control of society, they will bring about the most balanced and stable possible state […].”

This is particularly true for sociology’s positivistic and pragmatist branches, which Adorno addressed in his remarks, and which lend themselves particularly well to mechanistic thinking. It is, in this context, curious to find that mechanisms were used as theorizing devices by positivists like Durkheim and pragmatists like Addams and Mead but not by Marx and Engels. The latter invoke the term mechanisms mainly in the context of describing the mechanization of labor and the dehumanization of the worker by machines in factories, but they do not use the word to theorize the capitalist mode of production or the exploitation of the worker. In the spirit of Marx, the theorists of the Frankfurt School have consistently warned that a purely positivistic social science with its mechanistic worldview can dehumanize human beings by transforming them into quantitative data which makes them anonymous and disposable (just like workers under the capitalist mode of production). When we take this risk of dehumanization through science seriously, we can see that scientific mechanisms can introduce a distance between the researcher and the individual who in the eyes of the mechanicist is transformed from a holistic human being into an accumulation of interacting variables and from there into an engineering problem which, historically, has in the worst cases led to quite dehumanizing ideas like eugenics. But we can also draw attention to today’s self-help and optimization-culture with its regular calls to improve and perfect human beings with the help of a therapeutic outlook and scientific training of the body and the mind (Illouz, 2008; Nehring and Röcke, 2023). This culture of self-help and optimization, which encourages individual improvement through therapeutic tools and scientific knowledge, often proposes rapid solutions for personal development and individual efficiency by advocating the adoption of new behavioral habits and routines modeled after the principles of cognitive-behavioral therapy. In this context, the mechanistic view of the human body and mind not only influences scientific and medical perspectives but also extends its impact to broader societal attitudes toward the self and both accelerates and legitimizes further societal rationalization as it manifests instrumental reason as one of its guiding principles. With a mechanistic science being one of the dominant modes of thinking in the rational society, its premises pervade the collective consciousness and become a taken-for-granted stock of shared knowledge about how the physical and social world, the body, and the psyche work. Cognitive-behavioral therapies are the gold standard not only because they show their superiority through science, but because, from the point of view of Berger and Luckmann (1967), the rationalized society’s scientific institutions and common-sense are rigged in their favor. Their mechanistic outlook and positivistic framework speak to what is perceived to be self-evident truth about how the world works, a self-evident truth that is itself the product of an ongoing rationalization process and reproduced through scientific jargon. In such a rationalized society, mechanisms sell better on the marketplace of ideas as they implicitly confirm the knowledge-biases of scientific and everyday common-sense.

## 4 Conclusion

All of this goes to show that mechanisms, understood as the opening of a processual black box to see its discrete parts interact, are not merely neutral tools for understanding, they are actively creating and shaping the social world that we inhabit. While the manifest function of sociological theory may be to understand the world better, one of its latent functions is the creation of social worlds. Theories are not merely value-free tools for understanding, they are embedded in a cultural and historical context and project an intellectual and political milieu, such as the ideology of continual improvement, on to the social world. They are part of what Husserl (1970) called the *lifeworld*, a taken-for-granted stock of (tacit) knowledge that is uncritically invoked as we try to understand our surroundings and has a natural givenness, even though it is historically (i.e., socially) produced. According to Marcuse (2002, p. 166), Husserl “emphasizes the extent to which modern science is the ‘methodology’ of a pre-given historical reality within whose universe it moves.” Sociologists should indeed be fully aware of this given the success of phenomenology in the discipline. But even classics like Durkheim and Mauss had already argued that scientific categories are essentially and primarily social categories and, more recently, the cognitive sociologist Zerubavel (1991, p. 32) reiterated that mental reality is “deeply embedded in social reality.” This means that mechanisms are not only tools for scientific analyses, they (re)produce worldviews and, in so doing so, are both representing social realities (Fuhse, 2022), but also, as Bourdieu (2018) insists, “schemas for constructing reality.” In short, a scientific notion like the mechanism is not merely a matter of explanatory jargon, it pervades commonsense from where it legitimizes certain institutions and practices over others and legitimizes society’s increasing rationalization. Critical sociologists should therefore neither treat nor accept mechanisms as a self-evident truth about the social world but as a socially produced habit of thought in the rational society. They should not blindly turn into agents of rationalization, and always take a critical stance toward the whole as they analyze its parts. Most of all, they should not uncritically reproduce a cultural fetish and rather, in the words of Horkheimer (2002, p. 143), withstand the “growing aversion to seeing the human bottom of nonhuman things.”

## Data availability statement

The original contributions presented in the study are included in the article/supplementary material, further inquiries can be directed to the corresponding author.

## Author contributions

DD: Writing – original draft.
